# Physical Activity Promotes Health and Reduces Cardiovascular Mortality in Depressed Populations: A Literature Overview

**DOI:** 10.3390/ijerph17155545

**Published:** 2020-07-31

**Authors:** Martino Belvederi Murri, Federica Folesani, Luigi Zerbinati, Maria Giulia Nanni, Heifa Ounalli, Rosangela Caruso, Luigi Grassi

**Affiliations:** Institute of Psychiatry, Department of Biomedical and Specialty Surgical Sciences, University of Ferrara, Via Fossato di Mortara 64a, 44121 Ferrara, Italy; flsfrc@unife.it (F.F.); zrblgu@unife.it (L.Z.); nnnmgl@unife.it (M.G.N.); nllhfe@unife.it (H.O.); crsrng@unife.it (R.C.); luigi.grassi@unife.it (L.G.)

**Keywords:** depression, physical activity, exercise, mortality, cardiovascular diseases, cardiovascular risk factors

## Abstract

Major depression is associated with premature mortality, largely explained by heightened cardiovascular burden. This narrative review summarizes secondary literature (i.e., reviews and meta-analyses) on this topic, considering physical exercise as a potential tool to counteract this alarming phenomenon. Compared to healthy controls, individuals with depression consistently present heightened cardiovascular risk, including “classical” risk factors and dysregulation of pertinent homeostatic systems (immune system, hypothalamic–pituitary–adrenal axis and autonomic nervous system). Ultimately, both genetic background and behavioral abnormalities contribute to explain the link between depression and cardiovascular mortality. Physical inactivity is particularly common in depressed populations and may represent an elective therapeutic target to address premature mortality. Exercise-based interventions, in fact, have proven effective reducing cardiovascular risk and mortality through different mechanisms, although evidence still needs to be replicated in depressed populations. Notably, exercise also directly improves depressive symptoms. Despite its potential, however, exercise remains under-prescribed to depressed individuals. Public health may be the ideal setting to develop and disseminate initiatives that promote the prescription and delivery of exercise-based interventions, with a particular focus on their cost-effectiveness.

## 1. Introduction

Depression is associated with a shortened life expectancy, but this phenomenon might be counteracted by exploiting the numerous benefits of physical exercise. In recent years, research made us aware of a disconcerting finding: individuals who receive a diagnosis of a major depression live on average ten years less than non-depressed subjects [1]. This phenomenon has been largely attributed to the impact of physical diseases, rather than suicide or accidental deaths [2]. In particular, cardiovascular diseases seem to be responsible for the largest quota of premature mortality [3]. This is relatively unsurprising, given that several lines of evidence associate depression with increased cardiovascular risk at the population level [4]. Above all, depression itself is considered a risk factor for myocardial infarction and coronary death [5]; in addition, the American Heart Association included it as the only “psychosocial” risk factor among various indicators of adverse outcomes for individuals with acute coronary syndrome [6].

Several lines of evidence actually show that multiple, biologically plausible mechanisms may pave the way from depression to cardiovascular diseases. To mention the most robustly replicated findings, depression is associated with altered activity of the autonomic nervous system (ANS) of the heart [7], as well as with altered levels of pro-inflammatory markers [8,9].

The premature mortality of depressed individuals, overall, should not just pertain the field of psychiatry but also constitute a public health concern, considering the high prevalence of this disorder. According to a recent report, in fact, 4.4% of the world’s population suffers from a clinically relevant depressive disorder [10]. This alarming figure should make the depressed population a priority target for public health strategies to prevent cardiovascular mortality [2], and, based on several lines of evidence, interventions based on physical activity or physical exercise may be particularly fit to tackle this issue [11,12], while in the meantime they also improve the mental health of patients suffering from depression [13,14].

This narrative review has the aim of summarizing recent secondary literature (i.e., reviews and meta-analyses) that examines: (1) the cardiovascular risk and mortality of individuals suffering from depression; (2) the effects of physical activity or physical exercise on cardiovascular risk and cardiovascular mortality. We will conclude by briefly discussing commonly held misconceptions that may prevent clinicians from prescribing physical activity to depressed patients and potential implications for public health.

## 2. Depression Increases Mortality

Several epidemiological studies have shown that depression is associated with premature mortality. The effect of depression on mortality was specifically examined by a recent meta-analysis pooling the results of 293 studies, with an observation period ranging from less than 1 year to more than 10 years. Compared with subjects from the general population, depressed individuals had an unadjusted relative risk (RR) for mortality of 1.64 (95% CI 1.56–1.72) over the study period [15]. Several studies accounted for the role of different confounders, such as demographic variables, smoking, exercise, weight, and severity of the comorbid disorder. When only adjusted estimates were considered, the relative risk was lower, but still considerable (RR 1.52; 95% CI = 1.45–1.59). Results of single-cohort studies may also be informative of the impact of depression on mortality: among 5,103,699 Danish residents who were followed up from 1995 to 2013, those with depression displayed a double mortality rate compared to non-depressed subjects, corresponding to a shorter life expectancy by 14.0 years for men and 10.1 years for women [1]. Interestingly, while depression is more frequent among women, the phenomenon of premature mortality has been mostly observed among males [16].

The relationship between depression and mortality, however, is complex and likely to be influenced by several factors. Socioeconomic status, for instance, heavily impacts on health and could be partly responsible of the observed association [17]. Nonetheless, depression has been found associated with increased mortality both in low- and high-income countries [18].

The presence of physical diseases is another fundamental factor to consider when examining the association between depression and mortality. Indeed, physically ill individuals are expected to be frequently depressed, as well as display shortened life expectancy. In other words, observing a significant association between depression and mortality may not necessarily entail a causal relationship, since depression may constitute a mere consequence of the concomitant physical diseases (confounding by indication) [19]. Overall, cardiovascular diseases are of particular interest, since they are the leading cause of mortality in the general population, accounting for approximately one-third of all deaths [20], as well as among depressed subjects [19]. Available data suggest that the additional presence of depression in populations with cardiovascular diseases does indeed explain an increase in mortality, even when the severity of the primary disease is accounted for [15,21,22]. However, caution is required before interpreting such relationships as strictly causal [19]. Indeed, the severity of depression might be important to determine the prognosis of comorbid physical diseases. Even though mortality rates appeared to be higher among subjects with major, rather than subthreshold depression, this difference may not be meaningful [23].

In conclusion, the association between depression and premature mortality is a fairly robust epidemiological finding, albeit it might not necessarily entail a direct causal relationship [19].

## 3. Which Mechanisms Are Involved in the Higher Cardiovascular Risk of Depression?

Cardiovascular diseases are the main cause of premature death in depressive disorders, with major depression being associated with a hazard ratio of cardiovascular mortality of 1.63 (95% CI: 1.25–2.13) according to a recent meta-analysis [24]. More specifically, depression is as an independent risk factor for myocardial infarction and coronary heart disease [25] as well as coronary death [5], sudden cardiac death and the recurrence of arrhythmias such as atrial fibrillation [26]. Several lines of research have attempted to disentangle the multiple mechanisms that may mediate this phenomenon. Not surprisingly, the higher cardiovascular mortality rates observed among depressed subjects appear to be multi-factorial, involving biological as well as psychosocial factors. However, several of them appear to be amenable to modification.

### 3.1. Biological Factors

Subjects with major depression are prone to develop nearly all “traditional” cardiovascular risk factors, as illustrated in Table 1. Major depression is associated with a higher probability of becoming overweight and developing obesity since adolescence, especially among females [27,28], as well as a higher prevalence of type II diabetes [29], and hypertension [30]. Eventually, a higher prevalence of metabolic syndrome, hyperglycemia and hypertriglyceridemia was found in depressed individuals than control subjects in a meta-analysis of 18 studies [31], even controlling for confounding variables such as age, gender, and smoking habit. Surprisingly, however, discordant findings were observed in terms of serum lipid profile in depressed individuals: depression appeared to be associated with lower serum LDL when considered as a continuous measure and higher serum LDL when considered as a categorical measure [32]. The authors suggested the possibility of an association with lower LDL levels at the depression onset, with an increase in LDL over the course of the disease, in association with weight gain and metabolic syndrome.

Depressed individuals display a dysregulation of homeostatic systems [4]. In particular, dysregulation of the HPA axis is one of the most consistent biological alterations associated with depression [33], particularly in old age [34], with possible differences according to the melancholic or atypical subtype [35]. Depressed individuals display dysfunctions of the Autonomic Nervous System (ANS) in terms of a lower heart rate variability (HRV) that has been considered as a reliable predictor of negative cardiovascular outcomes [7,36]. The activity of the immune system is also abnormal in major depression, usually featuring an exaggerated pro-inflammatory state, both in the periphery and central nervous system [37,38,39,40,41]. Cross-sectional studies show that compared to healthy controls, depressed individuals consistently display higher levels of specific cytokines and chemokines in the peripheral blood, even after excluding physical comorbidities known to affect inflammatory markers [9]. A dysregulated immune system activity may derive from genetic predisposition [42] as well as altered neuro-immunological mechanisms [43,44,45] and behavioral factors such as tobacco use, thus the direction of the causal relationship between inflammatory markers and depressive symptoms appears to be extremely complex and likely bidirectional. Recent studies, for instance, suggest that abnormal TNF-alpha levels may directly cause the onset of depressive symptoms in adolescents [46]. Thus, depression and cardiovascular health have a complex, intertwined relationship. Both dimensions are regulated by the products of various pleiotropic genes involved in metabolism, HPA axis activity, inflammation, neurotransmission and circadian rhythms [47]. Animal models have been particularly useful to study the mechanisms underlying the comorbidity between depression and cardiovascular diseases and may provide further indications on their interplay. Recent evidence points to a role of chronic social stress as a trigger for depression, mediated by ANS dysfunction, endothelial damage and increases in pro-inflammatory cytokines [48,49].

### 3.2. Psychosocial Factors

People affected by depression are more prone to engage in unhealthy lifestyles, which in turn play a role in the pathogenesis of cardiovascular diseases. Depression appears to be associated with a tendency for unhealthy diets [50], and a higher risk of alcohol use disorder, in a bidirectional way [51]. Depression in adolescents (13–19 years old) predicts the taking up of cigarette smoking [52]; in turn, a history of depression is associated with lower probabilities of short- and long-term smoking cessation [53]. Depressed individuals also show a lower adherence to medications, especially for chronic disease such as cardiovascular ones [54]. In the elderly, the role of depression and physical diseases, especially cardiovascular disease, in impacting level of functioning and disability [55] is also mediated by low physical activity and time spent watching TV [56].

Last but not least, one of the most important and modifiable cardiovascular risk factors is physical inactivity. Compared with healthy individuals, those with depression are less likely to engage in physical activity and display a greater tendency of sedentary behaviors [12]; they also show reduced physical fitness [57], which is independently associated with cardiovascular events [58,59]. Recently, a large cross-sectional study, conducted on 1,237,194 adult people from the US confirmed the finding of a robust, U-shaped association between engagement in physical exercise and mental health in general, which held with adjustment for a number of sociodemographic and physical variables [60]. The authors observed that better mental health was associated with practicing specific sports, especially team sports, and it was proportional to the frequency and intensity of physical exercise, being worse at the extremes of minimal and excessive physical activity. A recent large study from Scotland on patients aged ≥ 16 years with cardiovascular diseases observed that meeting physical activity levels as recommended by the UK Chief Medical Officer was associated with better mental well-being [61].

## 4. Physical Activity May Narrow the Mortality Gap of Depression

**Figure 1:**
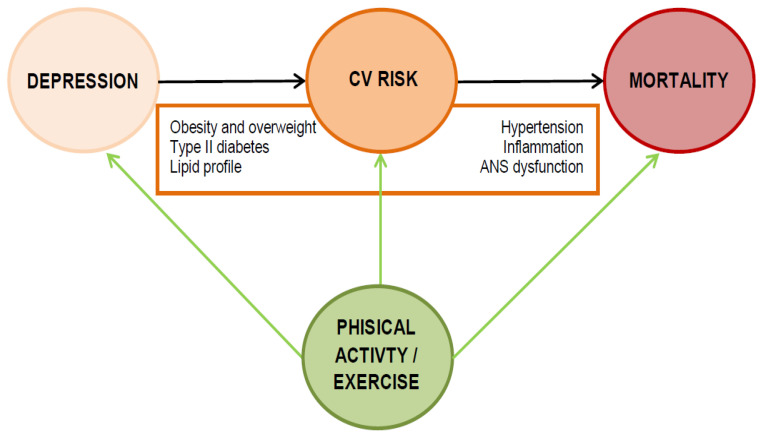
Effects of exercise in the relationship between depression, cardiovascular risk and mortality. ANS: Autonomic Nervous System.

Targeting physical inactivity may be one preferential strategy to narrow the mortality gap associated with depression. Several lines of evidence directly or indirectly support this view. This modifiable risk factor may be even the key for public health interventions [62,63]. Engaging in physical activity in depressed individuals, in fact, may not only have a direct favorable impact on the premature mortality rate associated with the disease, by reducing cardiovascular risk and cardiovascular mortality [64,65], but may also directly treat symptoms of depression [66]. Here, we make an approximation by conflating studies examining physical activity with those evaluating physical exercise, but we encourage potential readers in gaining a more detailed insight into their different implications. The former is a structured form of physical activity with a specific purpose directed toward physical health, while the latter is a generic form of activity [67]. Figure 1 depicts the effects of exercise in the relationship between depression, cardiovascular risk and mortality.

Several meta-analyses have evaluated the role of exercise on cardiovascular risk factors (Table 2). First, exercise improves cardiorespiratory fitness at different ages [68,69], even in the elderly [70] and when performed in a reduced-time session of moderate activity [71].

Second, exercise helps in weight loss together with a balanced diet, and counterbalances the negative effects associated with overweight and obesity [72,73,74]. These effects appear to be related to the influence of exercise on the metabolic profile associated with obesity and overweight: physical activity reduces fasting insulin levels [69,75] and visceral adiposity [76]. The latter, especially, is a strong predictor of morbidity and mortality, and exercise alone has proven to be more effective than a hypocaloric diet alone in weight reduction. An exercise session, moreover, acutely influences the levels of appetite-regulative hormones [77], even though its long-term effects are not yet been examined in detail.

Third, exercise contributes to achieve better glycemic metabolism in type II diabetes, as it reduces insulin resistance and HbA1c levels [69,78,79,80]. Physical activity also ameliorates the lipid profile: it reduces triglyceride levels, while increasing HDL levels [69].

Fourth, blood pressure control is better achieved with exercise [81,82,83], as it reduces both systolic and diastolic blood pressure in pre- and hypertensive subjects.

Fifth, physical activity normalized body homeostatic system activity. It modulates immune responses: exercise decreases C-reactive protein (CRP) and IL-6 levels in type II diabetes [84], and reduces CRP and fibrinogen levels in patients with coronary artery disease [85]. Furthermore, regular physical activity appears to benefit the immune system, slowing down its aging over time [86].

The ANS is also influenced by exercise, which improves HRV in type II diabetes [87], and cardiovascular diseases such as coronary artery disease and heart failure [88,89,90], suggesting an improvement effect on parasympathetic activity and a reduction effect on sympathetic activity. Further, the HPA axis, the main hormonal stress-response system, is influenced by physical activity: regular exercise, in fact, appears to ameliorate the stress response accelerating the return to a resting state [91,92,93].

The impact of structured physical activity on behaviors at risk for cardiovascular diseases was also examined in different studies; however, no effects on alcohol use nor smoking habit were observed [94,95] and the effects on sedentary behaviors appeared to be uncertain [96,97,98].

Cardiac rehabilitation (CR) represents an evident example of how useful exercise is for the secondary prevention of cardiovascular diseases: CR consists of integrated programs comprising physical, psychological and social interventions, with exercise playing a pivotal role. In particular, CR among coronary heart disease patients was effective reducing cardiovascular mortality (RR 0.74, 95% CI 0.64–0.86), reducing the long-term risk of myocardial infarction (RR 0.67, 95% CI 0.50–0.90), and the need for rehospitalizations (RR 0.82, 95% CI 0.70–0.96) [99]. Several guidelines on CR have been provided over the years, with notable differences across countries: nonetheless, the central element of CR seems to be aerobic endurance training [100].

The role of physical activity on mortality was estimated by a recent systematic umbrella review: physical activity reduced the risk of all-cause mortality (hazard ratios between 0.6 and 0.7), according to the intensity and frequency [101]. Moreover, significant effects were observed specifically on cardiovascular mortality, and even exercising at a lower intensity than recommendations by guidelines [101]. Resistance training was also associated with significant reductions in all-cause mortality (HR: 0.79; 95%CI: 0.69–0.91) but not cardiovascular mortality [102]. Overall, the impact on mortality in specific conditions such as coronary heart disease, stroke, heart failure, and diabetes was assessed as comparable to that of first-line medications [11].

An important additional effect of physical exercise on mortality might depend on its favorable effects on symptoms of depression. This has been observed in patients with cardiovascular diseases associated with depression. For instance, exercise was effective at reducing depression in patients after acute coronary events [103] and older adults with cardiovascular diseases [104]. In cardiovascular populations, evidence suggests the effectiveness of both high-intensity training [105] and low-intensity practices based on stress reduction, meditation or muscle relaxation [106]. However, part of the effects of exercise may depend on improving feelings of energy and fatigue [107]. Moreover, evidence supporting the antidepressant effect of exercise is rapidly growing in populations with primary major depressive disorder [108,109,110,111,112,113]. Studies are providing methodologically robust evidence on the efficacy of exercise alone for mild and moderate depression, and together with other treatments for severe depression [13,114,115]. The effects of depression on cardiovascular health, in fact, seem also dependent on the severity of depressive symptoms. By influencing the course and severity of depression, exercise may thus indirectly dampen such negative impact. In addition, it is still unknown whether exercise might actually impact on alcohol and substance use and cigarette smoking in mental health populations [116,117,118,119].

Finally, research is starting to investigate whether depressed patients undergo similar mechanisms of cardiovascular risk reduction that are observed in the general population and in cardiac patients. For instance, scheduling exercise sessions during Cognitive Behavioral Therapy was shown to positively modulate the immune system in depressed patients [120]. Similarly, cortisol levels appear to return to counteract HPA axis dysregulation in depressed patients [121]. Other improvements in neuroendocrine activity, autonomic nervous system activity, inflammatory markers and oxidative stress have been replicated in depressed populations, although further research is still needed before drawing firm conclusions [122,123,124].

## 5. How to Prescribe Physical Activity to Depressed Individuals

Physical activity has been included as a treatment for depression in the context of some clinical guidelines for depression, although its importance still remains downplayed for obscure reasons [13,125], whereas it is considered as an important treatment option to reduce mortality, especially mortality associated with cardiovascular conditions [126,127].

Despite the available evidence on its efficacy for depression, exercise remains under-prescribed. The reasons may be many, but among them, clinicians are often unaware of available indications on how to deliver physical activity to depressed individuals [128,129,130,131,132,133]. Actually, one of the first obstacles to overcome in order to improve the prescription of physical activity has been identified in physician prejudices and resistance based on the belief that patients will not adhere [13,134,135], whereas depressed patients usually display good adherence to exercise programs [136]; dropout rates from RCTs that include an exercise protocol are usually low and not different from those of control groups [115]. However, the presence of an instructor or other types of supervision may be crucial to motivate patients with severe mental illnesses to adhere to exercise programs [137]. Among depressed individuals, supervision is suggested at least in the initial phases [110]. Another reason why physicians may be reluctant to prescribe exercise to depressed individuals might depend on the perception that insufficient evidence is available on its efficacy, or on difficulties identifying the right “dose” to indicate to patients. In fact, historically, it has been difficult to identify a consistent threshold in terms of frequency and duration that achieves a meaningful reduction in cardiovascular disease incidence and mortality [13,101,138]. Moreover, the existing recommendations are mainly based on guidelines derived from the general or cardiovascular populations [114,139]. Some indications, however, may be translated to depressed populations in the absence of more specific data: cardiovascular benefits are immediately evident even adding small amounts of physical activity to the daily routine. Sedentary individuals, such as depressed patients, may display a steep risk decline even adding very short bouts (e.g., 10 min or less) of moderate–vigorous physical activity. Finally, reducing sedentary time or engaging in light physical activity also reduces cardiovascular risk, although it may require more time per day [138]. In sum, little physical activity is always “better than nothing” when it comes to cardiovascular risk reduction. Similar indications may become available regarding specific effects on mood and other depressive symptoms, with preliminary evidence suggesting that resistance and mixed training may yield higher efficacy than aerobic-only training [65].

By all means, however, the pleasure associated with exercise performances should be taken into account when prescribing physical activity [140]. In this so-called affect-based exercise, the goal of physical activity programs is mainly focused on the performance of activities associated with pleasant feelings, which may in turn also favor adherence to the exercise treatment. Ladwig and colleagues [140] suggest encouraging the patient to evaluate the pleasure associated with practice on a Likert rating scale regarding personal feelings, and then autonomously regulate the intensity and duration of exercise in order to maintain a satisfactory rating score on the aforementioned scale. Despite anhedonic experiences which are commonly observed in depression, these patients may still perceive exercise as pleasant [141,142,143]. The positive affective response obtained with exercise is also associated with treatment response, predicting both the improvement of depressive symptoms as well as the adherence to the exercise program [144,145].

Moreover, another barrier to the prescription and delivery of physical activity may depend on the need to involve different professionals and not necessarily physicians and other health professionals. However, for depressive and other mental disorders, it is highly recommended that the professionals involved have experience in the mental health field [146]. A collaborative and integrated approach with various disciplines is also highly recommended.

Taking the public health perspective, several interventions have been promoted to increase the physical activity level of the general population [62,63]. Some have proven effective, such as those involving telephone-assisted interventions, as well as changes in the workplace environment [138]. Furthermore, it has been observed that public health interventions for the promotion of physical activity have a high probability of being cost-effective in the general population [147] and among patients with mental disorders [148]. In this context, primary care might be a preferential setting to improve the physical activity habits of patients, benefiting especially patients with cardiovascular risk factors [149,150]. However, barriers limiting the prescription of exercise by clinicians need to be addressed. Some strategies deriving from behavioral economics have been provided to help overcome decision biases concerning physical activity [149].

### Limitations

This narrative review entails some limitations, the most evident being the lack of a systematic approach to the literature review. However, given the complexity of the topic and the heterogeneous methodological approaches (including epidemiology, mechanisms, as well as trial results on multiple outcomes), we deemed it useful to present the public health audience, as well as clinicians, with an overview of extant secondary literature, rather than focusing on more specific aspects.

## 6. Conclusions

The premature mortality of individuals with depression is a major unsolved issue not only for the mental health field, but also for public health. This phenomenon largely depends on a detrimental effect of depression on cardiovascular health, because this disorder leads to developing or exacerbating unhealthy lifestyles as well as causing imbalances across different body homeostatic systems. Among modifiable cardiovascular risk factors, physical inactivity may be the preferential target for clinical and public health interventions which may ultimately reduce the mortality gap. In fact, delivering physical exercise or physical activity may not only improve depression severity, but also directly tackle the constitutive elements of cardiovascular risk. Nonetheless, several challenges remain to be addressed by further research: (1) to provide more robust, direct evidence on the reduction in cardiovascular risk and mortality in depressed subjects; (2) to further tailor exercise- and physical activity-based interventions for depressed populations; (3) to extend the knowledge on, and tackle barriers to, exercise prescription by clinicians, and to provide them with streamlined indications to increase the prescription of exercise; (4) to assess the cost-effectiveness of exercise-based interventions; (5) to elaborate and assess public health strategies based on this effective, inexpensive and safe behavior.

## Figures and Tables

**Table 1:** Association between depression and cardiovascular risk factors.

Cardiovascular Risk Condition	Studies	Association between Depression and Risk Factor
Obesity and overweight	[27]	13 prospective studies on adolescents (of which, 7 evaluating depression leading to obesity and 6 obesity leading to depression). Bi-directional relationship, stronger for depression leading to obesity. Depression or depressive symptoms in adolescents is associated with an increased risk of 70% (RR 1.70, 95% CI: 1.40; 2.07) of becoming obese, while obesity in adolescents is associated with an increased risk of 40% (RR 1.40, 95% CI: 1.26; 1.70) of becoming depressed.
Type II diabetes	[29]	16 studies comparing major depressive disorder (clearly defined) to the general population in terms of the prevalence of type II diabetes. Major depression was associated with a higher risk for type II diabetes (RR 1.49; 95% CI = 1.29–1.72; *p* < 0.001) (when comparing age- and gender-matched populations: RR 1.36; 95% CI = 1.28–1.44; *p* < 0.001).
Metabolic profile	[31]	18 cross-sectional studies. Higher prevalence of metabolic syndrome in depressed (30.5%) than control individuals (OR 1.54, 95% CI = 1.21–1.97, *p* = 0.001); higher risk for hyperglycemia (OR 1.33; 95% CI = 1.03–1.73, *p* = 0.03) and hypertriglyceridemia (OR 1.17, 95% CI = 1.04–1.30, *p* = 0.008). Controlling for confounding factors.
	[32]	18 cohort studies. Lower LDL (mean difference = −4.29; 95% CI = −8.19, −0.40, *p* = 0.03) in depression when serum LDL considered as a continuous measure. Lower depression when low LDL (OR 0.90; 95% CI = 0.80–1.01, *p* = 0.08) when serum LDL considered as a categorical measure.
Hypertension	[30]	9 prospective studies, 22.367 participants, mean follow-up period 9.6 years. Increased risk of hypertension incidence with adjusted RR 1.42 (95% CI = 1.09–1.86, *p* = 0.009).
Inflammation	[9]	82 case-control studies. Elevated plasma levels of some cytokines and chemokines in depressed subjects (IL-6, TNF-α, IL-10, sIL-2R, CCL2, IL-13, IL-18, IL-12, sTINFR-2) (g = −0.477, *p* = 0.043).
Autonomic dysfunction	[7]	29 case-control studies. Lower HRV in depressed individuals (g = −0.349; CI 95% = −0.505, −0.193, *p* < 0.001).
	[36]	18 studies. Depression is associated with a lower HRV (g = −0.301, *p* < 0.001); negative correlation between depression severity and HRV (r = −0.354, *p* < 0.001).
**Behavioral Factors**		
Unbalanced diet	[50]	3 cross-sectional studies. 2 out of 3 studies support an association between depression and unhealthy diets.
Alcohol consumption	[51]	7 studies (2 out of 7 prospective studies). Increased risk of alcohol use disorder in depressed individuals (adjusted OR 2.09; 95% CI = 1.29–3.38).
Tobacco smoking	[52]	12 prospective studies. Depression predicted onset of smoking in adolescents (RR 1.41; 95% CI = 1.21–1.63, *p* < 0.001).
	[53]	42 clinical trials on smoking cessation. History of depression is associated with lower odds of short-term (OR 0.83; 95% CI = 0.72–0.95, *p* = 0.009) and long-term abstinence (OR 0.81; 95% CI = 0.67–0.97, *p* = 0.023).
Compliance to therapy	[54]	31 studies cross-sectional studies on chronic diseases. Depressed individuals are more often non-adherent to prescribed medications (OR 1.76; 95% CI = 1.22–2.57).
Sedentary behaviors	[60]	Cross-sectional study on more than 1 million individuals in US on mental health burden and its association with physical exercise.
	[12]	24 cross-sectional studies. Depressed individuals tend to engage less in physical activity (standardized mean difference = −0.251; 95% CI = −0.03, 0.15, *p* < 0.001) and more in sedentary behavior (standardized mean difference = 0.09; 95% CI = 0.01–0.18, *p* = 0.02).

**Table 2:** Effects of exercise on cardiovascular risk factors.

Cardiovascular Risk Factor	Studies	Impact of Physical Exercise
Obesity and overweight	[76]	117 studies. Exercise has better effects than a hypocaloric diet alone in reducing visceral adiposity (*p* = 0.08). However, it has less effects on total weight loss than diet alone.
	[77]	20 trials. Appetite-regulative hormone levels are acutely influenced by exercise.
Type II diabetes	[78]	27 prospective randomized or controlled trials of aerobic exercise training in adult subjects with type II diabetes, with a minimum duration of 2 weeks. Reduction in HbA1c% (mean difference = −0.71%; 95% CI = −1.11, −0.31, *p* = 0.0005) and insulin resistance (mean difference = −1.02, 95% CI = −1.77, −0.28, *p* = 0.007).
Lipid profile	[69]	160 RCTs. Exercise reduces triglycerides (*p* = 0.02), and increases HDL (*p* < 0.001).
Hypertension	[81]	93 RCTs. Reduction in systolic blood pressure and diastolic blood pressure. Different effects for different types of exercise and different blood pressure levels (greater for hypertensive patients).
Inflammation	[84]	14 RCTs. Exercise reduces CRP (−14% from baseline, 95% CI = −1.09, −0.23) and IL-6 levels (−18% from baseline, 95% CI = −1.44, −0.32) in type II diabetes.
	[85]	23 trials. Exercise reduces CRP (SMD = −0.500; 95% CI = −0.844, −0.157, *p* = 0.004) and fibrinogen levels (SMD = −0.544; 95% CI = −1.058, −0.030, *p* = 0.038) in coronary artery disease.
	[86]	Exercise enhances immune competency and slows down the aging of the immune system.
Autonomic dysfunction	[87]	15 trials. Improvements in HRV in type II diabetes after at least 3 month of an exercise program.
	[89]	16 RCTs. Exercise training leads to an improvement in HRV in coronary artery disease.
	[88]	19 studies (RCTs, quasi-RCTs and controlled trials of exercise training in adult patients with heart failure). Exercise improves HRV.

**Notes.** RCTs: Randomized Controlled Trials.
